# Surprising Effects of Ti and Al_2_O_3_ Coatings on Tribocatalytic Degradation of Organic Dyes by GaN Nanoparticles

**DOI:** 10.3390/ma17143487

**Published:** 2024-07-14

**Authors:** Xi Xu, Chenyue Mao, Jiannan Song, Senhua Ke, Yongming Hu, Wanping Chen, Chunxu Pan

**Affiliations:** 1 Key Laboratory of Artificial Micro- and Nano-Structures of Ministry of Education, School of Physics and Technology, Wuhan University, Wuhan 430072, China; 2023282020108@whu.edu.cn (X.X.); maochenyue1@163.com (C.M.); songjn@whu.edu.cn (J.S.); karson163163@163.com (S.K.); 2 Hubei Key Laboratory of Micro–Nanoelectronic Materials and Devices, School of Microelectronics, Hubei University, Wuhan 430062, China; huym@hubu.edu.cn

**Keywords:** tribocatalysis, coatings, dye degradation, GaN

## Abstract

GaN is more stable than most metal oxide semiconductors for the photocatalytic degradation of organic pollutants in harsh conditions, while its catalytic efficiency has been difficult to be substantially improved. In this study, the tribocatalytic degradation of organic dyes by GaN nanoparticles has been investigated. Stimulated through magnetic stirring using homemade Teflon magnetic rotary disks in glass beakers, the GaN nanoparticles were found to induce negligible degradation in rhodamine B (RhB) and methyl orange (MO) solutions. Surprisingly, the degradation was greatly enhanced in beakers with Ti and Al_2_O_3_ coatings on their bottoms: 99.2% and 99.8% of the 20 mg/L RhB solutions were degraded in 3 h for the Ti and Al_2_O_3_ coatings, respectively, and 56% and 60.2% of the 20 mg/L MO solutions were degraded in 24 h for the Ti and Al_2_O_3_ coatings, respectively. Moreover, the MO molecules were only broken into smaller organic molecules for the Ti coating, while they were completely degraded for the Al_2_O_3_ coating. These findings are important for the catalytic degradation of organic pollutants by GaN in harsh environments and for achieving a better understanding of tribocatalysis as well.

## 1. Introduction

The extensive use of fossil fuels by human society has not only consumed vast amounts of natural resources but has also generated huge amounts of exhaust gases, wastewater, and solid waste [1,2]. Due to the growing shortage of fossil fuels and environmental pollution, there has been an increasing focus on clean energy technologies to collect and utilize clean energies from the natural environment [3,4,5,6,7,8], such as thermal, solar, chemical, mechanical, and biological energies [9]. Photocatalytic oxidation, using semiconductors as catalysts and light as the energy source to degrade organic compounds, is an efficient, clean, and environmentally friendly technology [10]. To date, not only have various photocatalysts with novel catalytic performances emerged [11], such as Mie-resonant cuprous oxide particles with enhanced photocatalysis [12] and with tuned catalytic activity and selectivity [13], Fe-Cd co-modified ZnO with enhanced visible light photodegradation activity [14], PDMS−TiO_2_−Au sponge with highly enhanced photocatalytic activity [15], and nanostructured TiO_2_/ZnO heterojunctions with highly enhanced ultraviolet photocatalytic activity [16], but some valuable insights into the photo-redox mechanisms have also been revealed, such as the photocarrier recombination dynamics in Cu_2_O nanocatalyst clusters [17] and size- and shape-dependent charge-carrier dynamics in submicron cuprous oxide nanoparticles [18]. However, photocatalysis cannot operate beyond daylight hours, which significantly limits its practical environmental applications.

Apart from solar energy, mechanical energy is also a clean and sustainable energy in nature [19]. In recent years, there have been more and more papers on harvesting mechanical energy from nature for environmental remediation [2]. In 2019, Li et al. reported the tribocatalytic degradation of organic dyes using Ba_0.75_Sr_0.25_TiO_3_ (BST) nanoparticles under magnetic stirring [5], in which the BST nanoparticles absorbed mechanical energy through friction and converted it into chemical energy for degrading organic dyes. This was the first time that tribocatalysis appeared in environmental remediation. Previously, tribocatalysis was mainly investigated in the context of tribochemistry [20], especially for achieving ultralow friction and wear [21,22,23]. For environmental remediation, tribocatalysis does not require light irradiation within a specific intensity and wave-length range and thus has fewer environmental limitations than photocatalysis [24]. Tribocatalysis soon attracted great attention for environmental remediation, and, in the following couple of years, many materials, such as Bi_2_WO_6_ [25], BaTiO_3_ [26], FeS_2_ [27], Si [28], Ba(Zr_0.05_Ti_0.95_)O_3_ [29], NiCO_2_O_4_ [30], TiO_2_ [31], Fe_2_O_3_ [32], ZnO [33], SrTiO_3_ [34], CoFe_2_O_4_ [35], and CdS [36], have all been investigated for the tribocatalytic degradation of organic pollutants.

It is worth noting that some important photocatalysts, such as TiO_2_ [31], ZnO [33], and SrTiO_3_ [34], have also been employed in studies on the tribocatalytic degradation of organic pollutants. These metal oxides are well-known for their advantages of low cost, nontoxicity, and high chemical stability, which have made them important candidates in catalytic environmental remediation. With particles stimulated through magnetic stirring at relatively low rotating speeds (mostly around 400 rpm) in water, the heating effect in these studies has been mostly avoided, and a mechanism based on the excitation of electron–hole pairs in semiconductors by the mechanical energy absorbed through friction has been proposed for tribocatalysis [5], which is quite similar to that of photocatalysis. All these facts suggest that semiconducting materials are of special importance for the tribocatalytic degradation of organic pollutants. Together with numerous semiconducting metal oxides, Si as an elementary semiconductor and CdS as a typical II–VI semiconductor have also been investigated for the tribocatalytic degradation of organic pollutants and some interesting results have been obtained for them [36]. In contrast, no III–V semiconductors have been investigated for tribocatalytic applications to date.

With a band gap of 3.4 eV, GaN is an important III–V semiconductor due to its wide band gap, good chemical and thermal stability, and excellent optical and electrical properties [37,38,39]. Its high chemical stability makes it an ideal candidate, especially for the degradation of organic pollutants under extreme pH conditions, except that its photocatalytic efficiency has been difficult to be substantially improved through doping [40]. In our previous studies, we found that coating materials on the bottoms of beakers/reactors is an effective way to boost tribocatalysis [26,28,41,42,43]. Many materials have been studied as coatings for tribocatalysis, among which Ti and Al_2_O_3_ are very outstanding not only for their high chemical stability and surprising effects on tribocatalysis but also for their friendliness to the ambient environment: Ti is widely used as artificial bones to be implanted in human bodies, and Al_2_O_3_ itself is a component in soil. In this work, we have conducted an investigation on the tribocatalytic degradation of organic dyes by GaN nanoparticles. Although the GaN nanoparticles normally showed quite unsatisfactory tribocatalytic degradation efficiency for either the rhodamine B (RhB) or methyl orange (MO) solutions, the tribocatalytic degradation efficiency was found to be greatly improved through the Ti and Al_2_O_3_ coatings on the bottoms of the beakers. Moreover, two different types of MO degradation were observed between the Al_2_O_3_ and Ti coatings. These results should be relevant for GaN to be applied in the catalytic remediation of harsh environments and for better understanding tribocatalysis as well.

## 2. Materials and Methods

### 2.1. Materials and Characterization

A commercial GaN powder purchased from Shanghai Bide Pharmatech Ltd. (Shanghai, China) was investigated in this study. A commercial cubic boron nitride purchased from Shanghai Xingtian New Material Technology Co., Ltd. (Shanghai, China) (particle size 100 nm) was used for reference. Crystal structure was characterized through X-ray diffraction (XRD) using an X-ray diffractometer (BRUKER AXS D8 ADVANCE, Ettlingen, Germany) with Cu Kα radiation, and microstructure was analyzed using a field emission scanning electron microscope (SEM, Tescan CLARA, Brno, Czech Republic).

### 2.2. Forming Ti and Al_2_O_3_ Coatings on the Bottoms of Glass Beakers

Flat bottomed glass beakers with a dimension of φ 45 mm × 60 mm were used in this study. Ti and Al_2_O_3_ ceramic disks of φ 40 mm × 1 mm were first pasted on the bottoms of some beakers through a kind of strong glue (Deli super glue 502) separately. In this way, three kinds of beakers with glass, Ti, and Al_2_O_3_ bottoms were separately obtained.

### 2.3. Dye Degradation Tests

In a typical experiment, 300 mg of GaN powder were added into a glass beaker placed with a homemade Teflon magnetic rotary disk [26], followed by the addition of 30 mL of the 20 mg/L MO or 20 mg/L RhB solution. The suspension was then magnetically stirred at a speed of 400 rpm in dark, and the room temperature was kept at 25 °C. To monitor the degradation process, 3 mL of the suspension was sampled at fixed intervals, followed by centrifugation to collect the supernatant. The absorbance spectra were obtained using a UV–visible spectrophotometer (UV-2550, Shimadzu, Kyoto, Japan).

### 2.4. Detection of Active Species

For the detection of superoxide (·$O_{2}^{-}$) radicals, 10 mL of methanol (CH_3_OH), 0.15 g of GaN powder, and 0.05 mL of 5,5-dimethyl-1-pyrroline N-oxide (DMPO) were added into three glass beakers (φ 45 × 60 mm) with glass, Ti, and Al_2_O_3_ bottoms separately. For the detection of hydroxyl (·OH) radicals, 10 mL of deionized water, 0.15 g of GaN powder, and 0.05 mL of 5,5-dimethyl-1-pyrroline N-oxide (DMPO) were added into three glass beakers (φ 45 × 60 mm) with glass, Ti, and Al_2_O_3_ bottoms separately. Subsequently, the beakers were placed in dark at room temperature and stirred magnetically at 400 rpm using a homemade Teflon magnetic rotary disk for 15 min. The resulting suspensions were analyzed using an electron paramagnetic resonance (EPR) spectrometer (Bruker EPR A300-10/12) to identify the presence of superoxide (·$O_{2}^{-}$) and hydroxyl (·OH) radicals.

## 3. Results and Discussion

### 3.1. Materials’ Information

**Figure 1:**
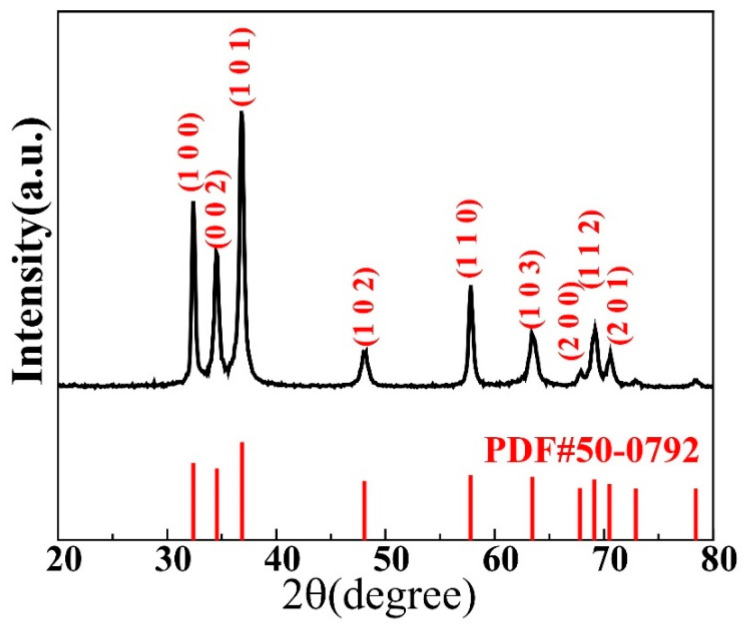
X-ray diffraction pattern taken for the GaN powder investigated in this study.

Figure 1 displays a typical XRD pattern obtained for the GaN powder used in this study. Most of the main peaks are sharp and of high intensity, indicating a high crystallinity. The peak positions in the graph match well with those of the standard PDF#50-0792 card of wurtzite GaN, as shown in the figure. No characteristic peaks of other impurities were observed, indicating the high purity of the GaN powder.

**Figure 2:**
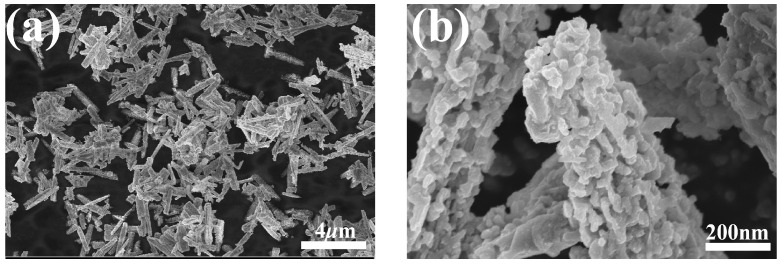
SEM micrographs of the GaN powder: (**a**) SEM micrograph at a low magnification; (**b**) SEM micrograph at a higher magnification.

Two representative SEM images of the GaN powder are shown in Figure 2. As shown in Figure 2a, under a relatively low magnification, the GaN powder is rods a couple of micrometers long, while, under a higher magnification, it can be clearly seen that the rods are actually aggregates of numerous nanoparticles, as depicted in Figure 2b. The nanoparticles are quite irregular in shape and coalesce into micrometers-long rods with tiny voids in them.

### 3.2. Tribocatalytic Degradation of RhB and MO

**Figure 3:**
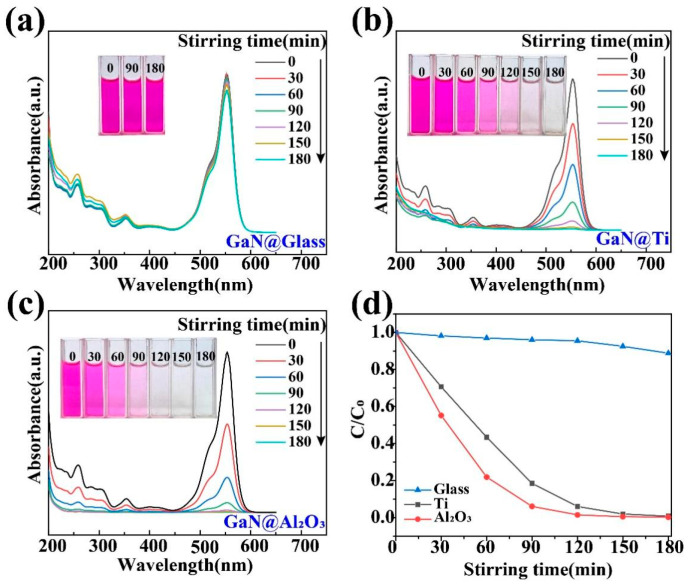
Tribocatalytic degradation of RhB (20 mg/L) solution by GaN nanoparticles characterized through UV–vis absorption spectra (inset: evolution of solution color): (**a**) GaN @ glass; (**b**) GaN @ Ti; (**c**) GaN @ Al_2_O_3_; (**d**) C/C_0_ vs. stirring time.

First of all, it should be pointed out that the GaN powder was found to show a very poor performance in the tribocatalytic degradation of the organic dyes in a normal way, namely in usual glass beakers. As shown in Figure 3a, after the GaN powder was stirred magnetically for 3 h in a glass beaker containing the 20 mg/L RhB solution, there was almost no change in the color of the RhB solution or in its absorbance of light, indicating a very low degradation efficiency. As a matter of fact, due to its relatively large band gap (3.4 eV), a very low photocatalytic degradation efficiency of the organic dyes was also observed for the GaN nanowires even under UV light irradiation [40]. It has been a great challenge to substantially increase the catalytic activity of GaN. Surprisingly, quite a different result was observed for the GaN powder when glass beakers with Ti and Al_2_O_3_ coatings were used, as shown in Figure 3b,c. In both cases, the RhB solution became colorless and the absorption peak at 554 nm disappeared after 3 h of magnetic stirring, indicating a degradation rate close to 100%.

To quantify the degradation efficiency of organic dyes, the formula D = 1 − C/C_0_ = 1 − A/A_0_ is used, where A and A_0_ represent the sustained and initial intensities of the characteristic absorption peaks of the dyes (RhB: 554 nm; MO: 464 nm), respectively. In this way, a more detailed comparison is shown in Figure 3d. After 3 h of magnetic stirring, only 3% of the 20 mg/L RhB solution was degraded in the glass beaker, while 99.2% and 99.8% of the RhB solution were degraded in the beakers with Ti and Al_2_O_3_ coatings, respectively.

**Figure 4:**
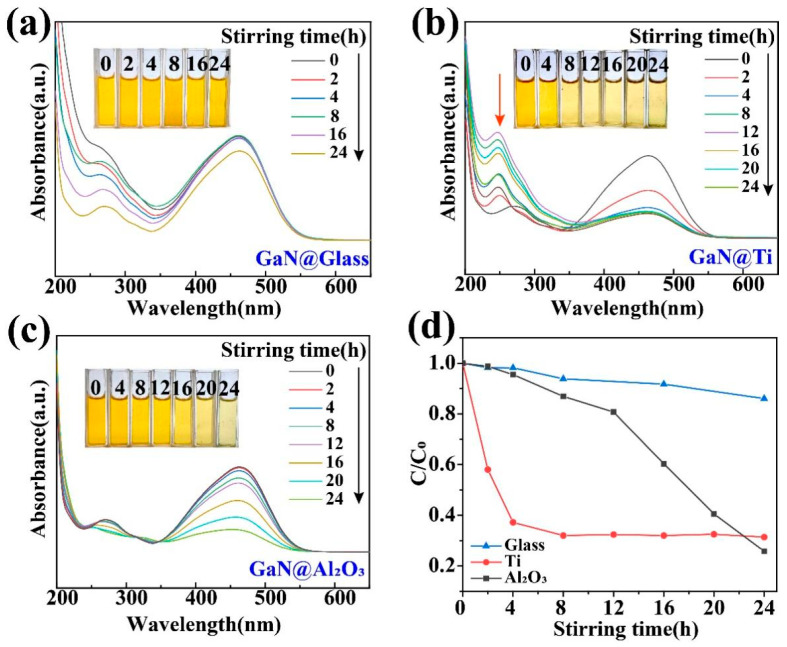
Tribocatalytic degradation of MO (20 mg/L) solution by GaN nanoparticles characterized through UV–vis absorption spectra (inset: evolution of solution color): (**a**) GaN @ glass; (**b**) GaN @ Ti; (**c**) GaN @ Al_2_O_3_; (**d**) C/C_0_ vs. stirring time.

The presence of high-energy bonds in dye molecules significantly affects the degradation process [29,30,35], which greatly increases the difficulty to degrade those dyes with high-energy bonds in them, such as MO. As shown in Figure 4a, in a usual glass beaker, the absorption peak at 464 nm in the UV–vis absorption spectra almost remained unchanged even after 16 h of magnetic stirring. In contrast, when glass beakers with Ti and Al_2_O_3_ coatings were used, the absorption peak decreased significantly after 24 h of magnetic stirring, as shown in Figure 4b,c. For a more detailed comparison, as shown in Figure 4d, when glass beakers with Ti and Al_2_O_3_ coatings were used, the degradation efficiency after 24 h of magnetic stirring is increased to 5 times and 5.3 times the ordinary glass beaker, respectively. More importantly, it is worth noting that the degradation of the MO associated with the Ti coating was quite different from that with the Al_2_O_3_ coating. For the former, a peak around 250 nm was formed in the course of the degradation process, while, for the latter, such a peak was not observed. As a matter of fact, these two types of MO degradations had been studied in detail in some previous investigations. The appearance of the absorption peak around 250 nm actually indicates that the MO molecules were only broken into some smaller molecules like benzoic acid, succinic acid, and p-phenol and the degradation was only a partial degradation [28,44,45]. Obviously, these by-products should still be regarded as organic pollutants in the ambient environments. Fortunately, such a peak did not appear when MO molecules were degraded mostly into H_2_O and CO_2_ for the Al_2_O_3_ coating. Such a contrast between the Ti and Al_2_O_3_ coatings not only highlights the advantage of the Al_2_O_3_ coating for GaN nanoparticles but also demonstrates the importance of the coating choice in tribocatalysis. It is worth noting that, for dynamic friction between two solid materials, their surfaces are put into intimate contact by a compressive force and strong interactions can occur between them through sliding. Accordingly, the tribocatalytic behavior of the GaN must have been affected by the coating materials in a very complicated way.

It has been a great challenge to improve the photocatalytic performance of GaN nanomaterials. While doping to GaN is rather difficult, forming composites/heterostructures with other materials, such as ZnO [46,47,48,49,50], has been widely adopted for GaN. It has to be pointed out that, however, these materials including ZnO are usually less stable in harsh environments than GaN, and their composites/heterostructures are not suitable for applications in harsh environments. On the other hand, more and more research has shown that, for tribocatalysis, coating materials on the bottoms of beakers is an effective and convenient method to achieve a better catalytic performance [26,28,41]. As for the Ti and Al_2_O_3_ coatings in this study, not only are these two kinds of materials chemically stable in harsh environments but also both disks for these two kinds of coatings are uniform in composition from the surface to interior, indicating that they can be adopted for GaN to achieve an improved degradation efficiency of organic pollutants in harsh environments and when some surface layer of the coatings is worn off after some prolonged service. As a matter of fact, both kinds of coatings had repeatedly been used in our laboratory and there were no detectable changes in their performance. It has to be pointed out that the tribocatalytic performance of GaN nanoparticles with Ti and Al_2_O_3_ coatings is still lower than that of many metal oxides [27,43]. The effects of more kinds of coatings on tribocatalysis should be further extensively explored for GaN nanoparticles.

### 3.3. Mechanism Studies

**Figure 5:**
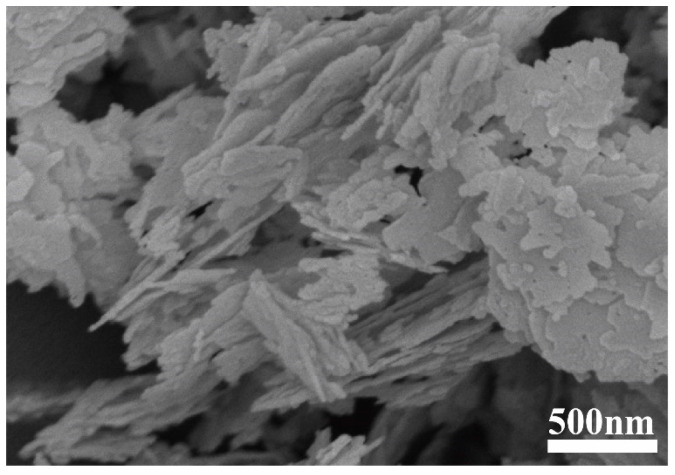
SEM micrograph of as-received BN powder.

**Figure 6:**
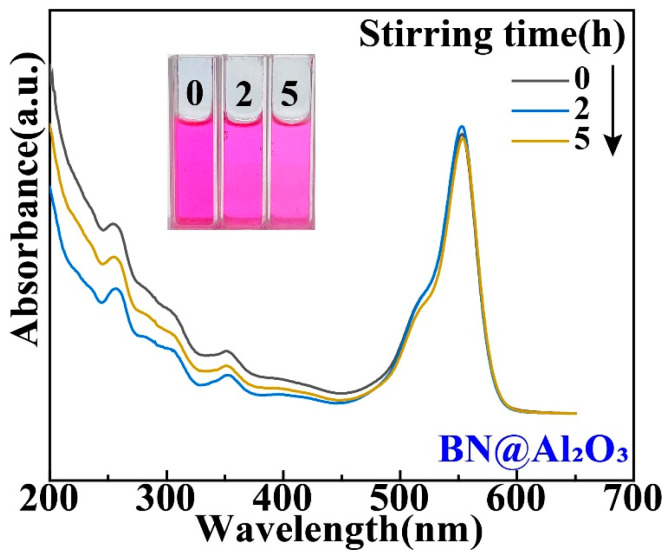
Tribocatalytic degradation of RhB (20 mg/L) solution by BN nanoparticles in a beaker with Al_2_O_3_ coating characterized through UV–vis absorption spectra (inset: evolution of solution color).

While the Ti and Al_2_O_3_ coatings were found to have surprising effects on the tribocatalytic degradation of organic dyes by GaN nanoparticles, it is necessary to examine whether the effects have mainly arisen from the coatings themselves [22]. For this purpose, we utilized a commercial cubic BN powder to replace the GaN powder as the catalyst. Figure 5 shows an SEM image of the cubic BN powder, which appears flake-shaped with a thickness around 30 nm. As shown in Figure 6, in a beaker with an Al_2_O_3_ coating, the RhB solution almost showed no degradation after the BN nanoparticles were magnetically stirred for 5 h, forming a stark contrast to the results obtained with the GaN nanoparticles. This phenomenon suggests that Al_2_O_3_ itself is chemically inert and the enhanced tribocatalytic degradation of the organic dyes by the GaN nanoparticles associated with the Al_2_O_3_ coating has resulted from the interaction, namely the friction, between the GaN nanoparticles and the Al_2_O_3_ coating.

A mechanism based on the excitation of the electron–hole pairs in semiconductors has been proposed for tribocatalysis [5]. Accordingly, for the GaN nanoparticles in this study, the tribocatalytic degradation of organic dyes can be expressed as follows:

(1) $$GaN\;NPs\overset{Friction}{\to }GaN\;NPs+h^{+}+e^{-}$$

(2) $$OH+h^{+}\to \hat{A}\cdot OH$$

(3) $$O_{2}+e^{-}\to \hat{A}\cdot O_{2}^{-}$$

(4) $$h^{+}/e^{-}/\hat{A}\cdot OH/\hat{A}\cdot O_{2}^{-}+RhB\to degradation$$

**Figure 7:**
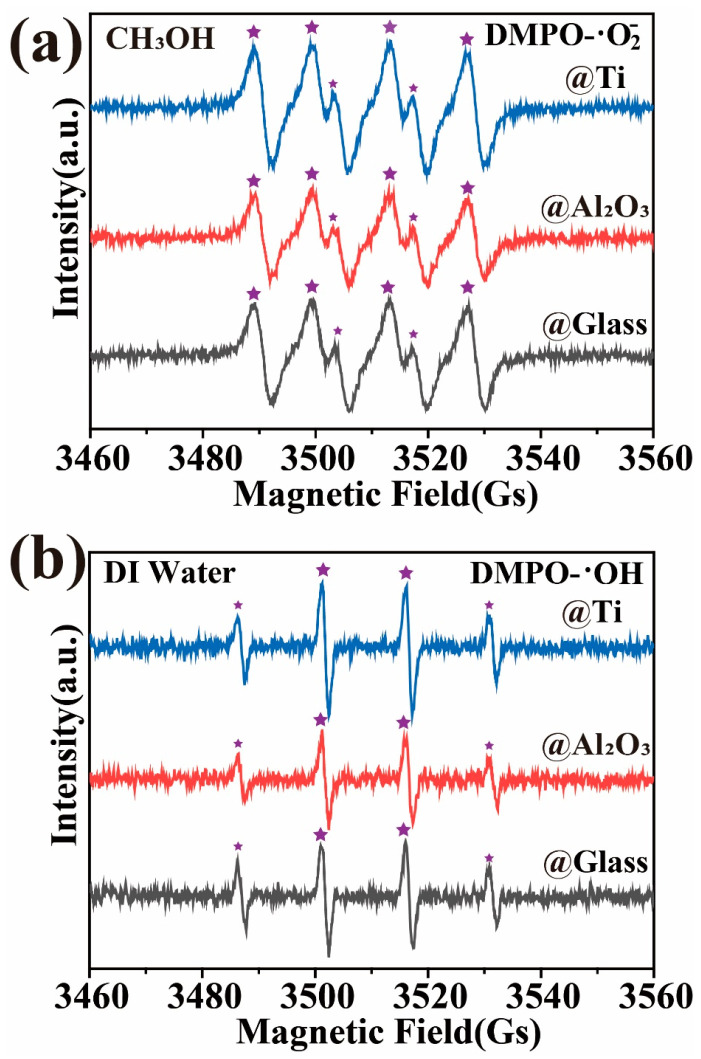
EPR spectra for three different coatings: GaN nanoparticles were magnetically stirred in deionized water and DMSO separately using a PTFE magnetic rotating disk.

The results of the EPR experiments are shown in Figure 7, where the signals corresponding to DMPO-·$O_{2}^{-}$ (Figure 7a) and DMPO-·OH (1:2:2:1, Figure 7b) can be observed during the magnetically stirred induction process of GaN nanoparticles in beakers with glass, Ti, and Al_2_O_3_ bottoms separately. The same results have also been reported in previous studies [5,30,51], which provide strong evidence for the excitation of electron–hole pairs by the mechanical energy absorbed through friction in semiconducting materials, including GaN. However, it has to be pointed out that, as DMPO-·$O_{2}^{-}$ and DMPO-·OH radicals were found to be similarly generated for the three kinds of bottoms, the EPR results provided no evidence for understanding the surprising effects of the Ti and Al_2_O_3_ coatings on the tribocatalytic degradations of the organic dyes by the GaN nanoparticles. More analyses are highly desirable in future investigations.

**Figure 8:**
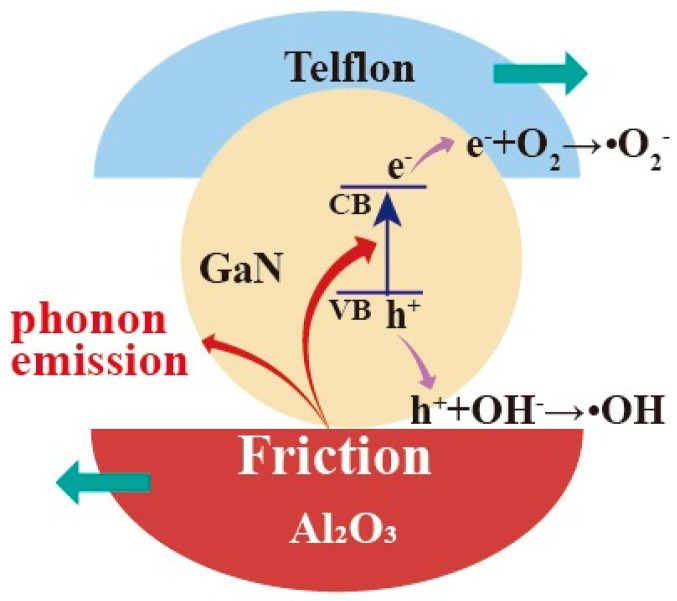
Absorption of mechanical energy by GaN nanoparticles through friction under magnetic stirring in a glass beaker with Al_2_O_3_ bottom and the excitation of electron–hole pairs in GaN.

For GaN nanoparticles under magnetic stirring in beakers, the friction between the GaN nanoparticles and beaker bottoms should be mostly responsible for the excitation of the electron–hole pairs in the GaN. In this way, the dependence of the tribocatalytic behavior of the GaN nanoparticles on the coatings observed in this study can be preliminarily explained. Among the dynamic frictions between the three friction pairs (GaN and glass, GaN and Ti, and GaN and Al_2_O_3_), the one between GaN and Al_2_O_3_, as shown in Figure 8, is most outstanding in that not only is the fastest degradation of RhB observed for the Al_2_O_3_ coating in Figure 3 but also a complete degradation of the MO can only be observed for the Al_2_O_3_ coating in Figure 4. Nevertheless, it is still a great challenge to fully understand the coating dependence of the tribocatalytic behavior of GaN nanoparticles.

## 4. Conclusions

Neither the RhB nor the methyl orange MO solutions were evidently degraded by the GaN nanoparticles under magnetic stirring using homemade magnetic rotary disks in glass beakers. Surprisingly, greatly enhanced degradation was obtained after the Ti and Al_2_O_3_ disks were coated on the bottoms of the beakers separately. For the Ti coating, 99.2% of the 20 mg/L RhB was degraded in 3 h and 56% of the 20 mg/L MO solution was degraded in 24 h. For the Al_2_O_3_ coating, 99.8% of the 20 mg/L RhB solution was degraded in 3 h and 60.2% of the 20 mg/L MO solution was degraded in 24 h. Moreover, a striking contrast was observed in the MO degradation between the two coatings: the MO molecules were only broken into smaller organic molecules for the Ti coating, while they were degraded into H_2_O and CO_2_ for the Al_2_O_3_ coating. These findings provide a practicable method to improve the catalytic efficiency of GaN for the degradation of organic pollutants in harsh environments and will enrich our understanding of tribocatalysis as a whole.

## Data Availability

The original contributions presented in the study are included in the article, further inquiries can be directed to the corresponding authors.
